# An Important Step in Neuroscience: Camillo Golgi and His Discoveries

**DOI:** 10.3390/cells11244112

**Published:** 2022-12-18

**Authors:** Vicentiu Mircea Saceleanu, Razvan-Adrian Covache-Busuioc, Horia-Petre Costin, Luca-Andrei Glavan, Alexandru Vlad Ciurea

**Affiliations:** 1 Neurosurgery Department, Sibiu County Emergency Hospital, 550245 Sibiu, Romania; 2 Department of Neurosurgery, “Lucian Blaga“ University of Medicine, 550024 Sibiu, Romania; 3 Department of Neurosurgery, “Carol Davila” University of Medicine and Pharmacy, 020021 Bucharest, Romania; 4 Neurosurgery Department and Scientific, Sanador Clinical Hospital, 70000 Bucharest, Romania; 5 Department of Medical Sciences, Honorary Member of the Romanian Academy, 010071 Bucharest, Romania

## 1. General Data

**Figure 1:**
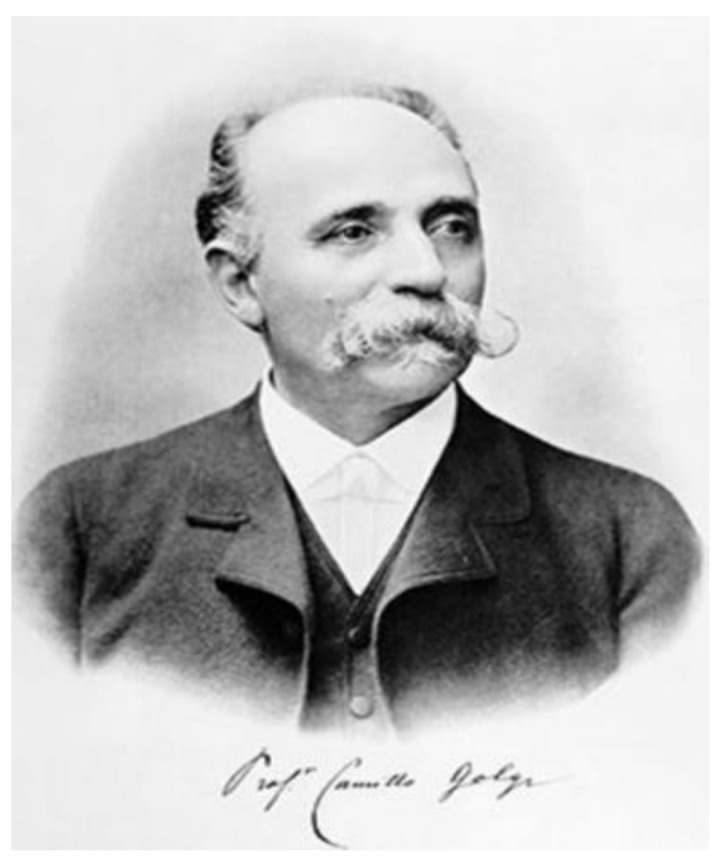
Picture of Camillo Golgi. Image in public domain and free from copyright issues.

Camillo Golgi (Figure 1) is one of the most prestigious personalities of modern medicine. His success is due to the revolutionary research he has conducted in fields such as cell biology, histology, anatomy, neurology, neuroscience and parasitology. Thus, his prestige is due to the numerous discoveries that support modern science.

He was born on 7 July 1843 in the town of Corteno in the province of Brescia, Italy. Inspired by his family, Camillo Golgi began his studies in medicine at the University of Pavia, finishing them in 1865 [1,2]. Since his father was also a medical doctor, the only purpose for Camillo Golgi was to help him; however, the general vision of Golgi concerning medicine was changed when he started his collaboration with his mentor, the doctor Cesare Lombroso (1835–1909), the founder of the “Italian school of positivist criminology”. Under his guidance, Camillo Golgi wrote his dissertation based on the etiology of mental illness [3], thus obtaining his medical degree in 1868. Thanks to this collaboration, Camillo Golgi was attracted by the desire to explore the human brain, a research area of interest, which was lacking many breakthroughs at the time.

Subsequently, due to numerous antithetical perspectives between Cesare Lombroso and Camillo Golgi, the collaboration between them ceased. This is when Camillo Golgi met a true personality of science at that time, Giulio Bizzozero (1846–1901), a well-known pioneer of histology, famous for his studies in the field of microbiology (helicobacter pylori) and for describing the platelets [3]. The collaboration was very beneficial for Camillo Golgi because Giulio Bizzozero mentored him in the field of optic microscopy, influencing his further research.

In 1872, Camillo Golgi became Chief of the Hospital of the Chronically Ill in Abbiategrasso, near Milan. This is where he invented, in 1873, the “Black Reaction” [4]. Later on, in 1879, he was appointed Chief of Anatomy at the University of Siena for one year. Returning to Pavia, he was appointed Professor of General Pathology and the honorary chief of the medical hospital in Pavia [5]. Camillo Golgi held the position of Rector of the University of Pavia two times: the first time between 1893–1896, and later on 1901–1909. The year 1918 marks his retirement, his research in his laboratory continuing until 1923. Despite an existence full of glorious and impactful achievements, Camillo Golgi quietly passed away at the age of 82, on 21 January 1926, in Pavia, the city where he spent a major part of his life.

## 2. Neuroscience Contribution

The two influences exerted by his mentors, Giulio Bizzozero and Cesare Lombroso, shaped his research emphasizing the structure of the nervous system. Governed by the question “What are the cytoarchitectonics of the fascinating nervous system?”, Camillo Golgi began research in this area.

In the 19th century, the general aspects regarding the morphology of the nervous cell were not entirely elucidated. Earlier that century, Otto Friedrich Karl Deiters (1834–1863) managed to demonstrate a certain continuity between the nerve cell and its extensions—axons and dendrites [6]. Additionally, the methods of fixation for the histological specimens at that time were inappropriate for capturing the pretentious nervous tissue under a microscope; hematoxylin or carmine did not provide satisfactory results, the reason being the extraordinarily small space between the cells of the nervous tissue.

That was the moment when, in the year 1873, Camillo Golgi made a revolutionary discovery, which totally changed the trajectory of the neurological field. Camillo Golgi was studying some specimens of nervous tissue, fixed according to a new technique. Small pieces of nervous tissue were kept in aldehyde, and later they were hardened in potassium dichromate. After this, silver nitrate reacted with pieces of the specimen that have been hardened in potassium dichromate. This coloration technique was later called the “Black Reaction”—“La Reazione Nera” in Italian, or, as it is now known in the scientific community, “Golgi’s impregnation”—“Golgi Staining”.

There are two advantages of using this technique: the first one is that the silver chromate will form microparticles inside the cells, while the second one is that the “Black Reaction” only impregnates just a couple of the cells inside the histological sample, in comparison with other methods. This is very useful, especially for studying the histology of the nervous system, as it allowed Golgi to easily differentiate axons from dendrites. Unlike the classical methods, the fixation by using solutions with metals determines the preferential precipitation on certain structures, through a mechanism that is not even to this day completely elucidated [7].

A year before the invention of the “Black Reaction”, Joseph von Gerlach (1820–1896), a German anatomist, formulated the reticular theory. It claims that the nervous system would actually function as a syncytium due to the multitude of dendritic ramifications, therefore it would, in fact, function as a whole [8]. Glial cells, particularly the astrocytes, create an enormous network that connects vast parts of the nervous system, by modulating and regulating synapses altogether.

On the other hand, the enunciation of the reticular theory also implies the description of its rival, “The Neural Theory” (or neuron doctrine), postulated by Santiago Ramón y Cajal. It assumes that the neuron functions as a unit in itself, not as an entire system. The statement is based on studies conducted by the Spanish researcher, who observed a discontinuity between neuronal extensions, later called synaptic [8].

Cajal was amazed by the quality of Camillo Golgi’s tissues impregnated using the Black Reaction method, and in fact, more than half of his sketches were made using this method. Moreover, Cajal even made certain improvements to the protocol [7]. Thus, in 1891, “The Neural Theory” was formulated by the Spanish researcher, a theory which denounces The Reticular Theory as being obsolete.

The debate between the two theories was the foundation of modern neuroscience, and for about a century, “The Neural Theory” was the central dogma of this field. For more than a century, the reticular theory was shadowed by the concept postulated by Cajal. However, it is important to state that glial cells were an underdeveloped subject, the spotlight being on the neurons and their morpho-physiological particularities. We believe that the truth is a comprehension of the best arguments presented by both theories, as numerous recent studies have revealed new information about the functioning of the nervous system. A functional syncytium created by gap junctions between the astrocytes has been identified. Astrocytes, in turn, can form gap junctions with oligodendrocytes as well. The bonds between the astrocytes are tightened at the level of the gap junction most often by the Cx43 protein [9]. This functional syncytium can be described, briefly, by an extrapolation: an astrocyte is in simultaneous contact with about 140 thousand nervous cells [10], and currently, it is considered that the number of astrocytes exceeds the number of neurons in the nervous system of mammals [11]. At the same time, linking these cells through gap junctions will decrease the electrical resistance between cells, allowing joint depolarization and creating a similar behavior for all the components of the system, functioning therefore as a singular unit [12]. The property of astrocytes to work as a syncytial unit represents a key point in synchronization of the nervous system network regarding the brain state [13].

**Figure 2:**
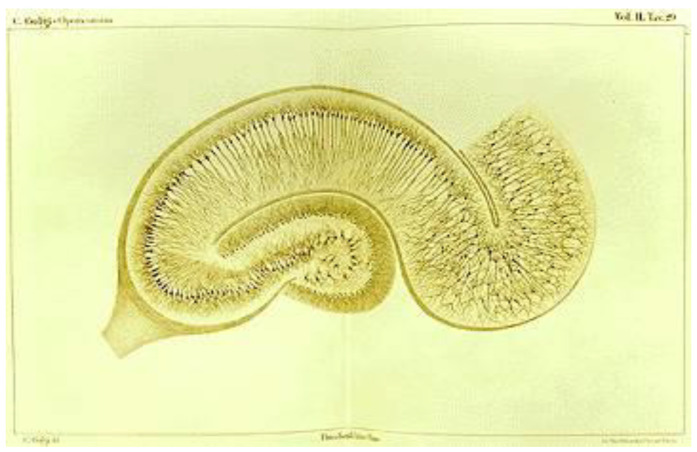
Figure showing microscopic drawing of hippocampus stained with Black Reactio. Histological plate prepared by Camilo Golgi. Image in public domain and free from copyright issues. Source of Image: Wikimedia Commons.

It is interesting to note that, in 1903, Camillo Golgi published a paper entitled “Opera Omnia”, a work in which numerous anatomical nerve structures were sketched. Among them are the cerebellum and the hippocampus (Figure 2), formations that present numerous glial cells, explaining this way the fierce support of the reticular theory by the Italian researcher [14].

## 3. Important Discoveries

On the basis of “The Black Reaction”, Camillo Golgi published in 1874 the work “Sulla fina anatomia del cervelletto umano” [15]. In this work, the researcher made a detailed description regarding the cytoarchitectonics of the nervous system, simultaneously with the description of a nervous cell that bears his name up to this moment: Golgi cell. The scientist noticed the presence of a developed axonal plexus, a criterion that is still used nowadays to observe a Golgi cell on a histological sample. In the same paper, Golgi outlines the hypothesis of a local interconnectivity, such as nervous cells functioning as a whole, enhanced and synchronized by the glial cells, which can be found in the entire nervous system. One of the functions of Golgi cells is that, being GABAergic and glycinergic, they inhibit neuronal circuits in the vestibulocerebellar loop [16].

In 1878, he discovered two new types of sensory receptors. First of all, he discovered the Golgi–Mazzoni corpuscles, that encapsulate the nerve ending, being fine pressure receptors that are located only in the fingertips. These structures are similar to the Vater-Pacini receptors, except for their localization. Second of all, another discovery, which took place in 1878, was called the Golgi tendon organ, located in the muscle–tendon junction and representing a muscular proprioceptor [3].

Starting in 1879, Camillo Golgi was appointed Professor of General Pathology at the hospital in Pavia, thus having the opportunity to study the in vivo evolution of malaria. By associating the clinical symptomatology developed by his patients with the evolution of the parasite in the blood, the researcher managed to elaborate a description of the cycle that takes place in the human erythrocyte regarding Plasmodium, now known as the Golgi cycle [17].

Later that century, Camillo Golgi made new discoveries regarding the kidney’s histology. He noticed that in each nephron, the ascending part of the Henle loop returns back to the cortical, juxtamedullary zone. This part comes in close contact with the Malpighi corpuscle, and was later called the juxtaglomerular apparatus, important in regulating blood pressure through renin secretion.

In 1898, while analyzing the Purkinje cells in the cerebellum, Camillo Golgi observed what we now call the Golgi apparatus, through silver nitrate staining. Immediately after this, he discovered that the Golgi apparatus could also be found inside the neural cells of the spinal ganglia. This event started an avalanche of adjacent research that proved the presence of the Golgi apparatus in numerous other types of structures. Initially, it was called “internal reticular apparatus”, due to the fact that, at that time, the function of the Golgi apparatus could not be stated, and it was assumed for a long time that the organelle was actually a fixation artifact. This whole situation was called “The Golgi Controversy” [18]. The dilemma was solved in 1954, when the first observation of this organelle was made through electron microscopy. Subsequently, the uneven division of the phosphatases on the trans face of the Golgi apparatus suggests its segmentation into several parts. Other studies have involved the use of the staining method used by the Italian scientist when describing the organelle and observing the specimens, this time using electron microscopy. The result was the discovery of the cis face of this organelle, which was the only part that was highlighted by this method [19]. This organelle was a central pillar of the Nobel lecture of researcher George E. Palade, in which he explained the mechanism of extracellular secretion, concerning the subject of the acinar pancreatic cell [20].

## 4. Recognition

Camillo Golgi’s prestige has been recognized by numerous international faculties and scientific societies. They paid tribute to the scientist by choosing him as Doctor Honoris Causa of the Universities of Cambridge, Athens, Paris (Université de la Sorbonne), Geneva and Kristiania (Oslo), also being a Nobel laureate in 1906, along with Santiago Ramón y Cajal, an award obtained in recognition of their work regarding the structure of the nervous system.

Additionally, both scientists held Nobel lectures that summarized their findings: Santiago Ramón y Cajal: “The Structure and Connexions of Neurons” and Camillo Golgi: “The neuron doctrine—theory and facts”.

## 5. Conclusions

Despite the fact that Santiago Ramón y Cajal is proclaimed as the father of modern neuroscience because he promoted “The Neuron Doctrine”, we believe that researcher Camillo Golgi deserves at least as much recognition. Numerous recent studies justify the return of the reticular theory in the spotlight of neuroscience, bringing it back to relevancy.

If Santiago Ramón y Cajal’s work is a marble statue, Camillo Golgi’s work was the podium of the statue, but also the marble block from which it was carved. “La Reazione Nera”, through the silver impregnation of nervous structures is the testament of Golgi’s genius, the gold standard for neuroscience research.

## Data Availability

Not applicable.
